# Supplementary material for: Complexome profiling of the Chlamydomonas psb28 mutant reveals TEF5 as an early PSII assembly factor
Source: Plant Cell. 2025 Mar 17;37(6):koaf055. doi: 10.1093/plcell/koaf055 (PMC12164590; doi:10.1093/plcell/koaf055)
Supplement: koaf055_Supplementary_Data [file koaf055_supplementary_data.zip › psb28_interactive.zip!/plots/intensity/qualityPlots/peptides/BIO1_Cre06.g277200.t1.2.html]

### Cre06.g277200.t1.2

Trivial name: BIO1  
SeqCoverage [median]: 5.00  
Mass: 110.22 kDa  
  
 

| Sequence | MissedCleavage | Mass [Da] | wtIntensity | muIntensity | Score |
| --- | --- | --- | --- | --- | --- |
| ASGAATATAGAAAAAATAAPGR | 0 | 1755.89 | 0.00 | 416.81 | 46.95 |
| AVSDEQLVVATGR | 0 | 1343.70 | 0.00 | 382.80 | 89.40 |
| GPGAGWAER | 0 | 899.42 | 0.00 | 1831.99 | 62.66 |
| YAAVSSAAVVR | 0 | 1092.59 | 0.00 | 323.91 | 66.62 |
|  |  |  | 0.0 | 2955.5 |  |
